# Oncobiosis and Microbial Metabolite Signaling in Pancreatic Adenocarcinoma

**DOI:** 10.3390/cancers12051068

**Published:** 2020-04-25

**Authors:** Borbála Kiss, Edit Mikó, Éva Sebő, Judit Toth, Gyula Ujlaki, Judit Szabó, Karen Uray, Péter Bai, Péter Árkosy

**Affiliations:** 1 Departments of Oncology, University of Debrecen, 4032 Debrecen, Hungary; bkiss@med.unideb.hu (B.K.); tothjuditdr11@t-online.hu (J.T.); 2 Departments of Medical Chemistry, University of Debrecen, 4032 Debrecen, Hungary; miko.edit@med.unideb.hu (E.M.); ujlaki.gyula@med.unideb.hu (G.U.); karen.uray@med.unideb.hu (K.U.); 3 Kenézy Breast Center, Kenézy Gyula County Hospital, 4032 Debrecen, Hungary; seboeva@gmail.com; 4 Medical Microbiology Faculty of Medicine, University of Debrecen, 4032 Debrecen, Hungary; szabjud@med.unideb.hu; 5 MTA-DE Lendület Laboratory of Cellular Metabolism, 4032 Debrecen, Hungary; 6 Research Center for Molecular Medicine, Faculty of Medicine, University of Debrecen, 4032 Debrecen, Hungary

**Keywords:** pancreatic adenocarcinoma, oncobiome, microbiome, bile acids, bacterial metabolite, amino acid metabolites, polyamines, LPS, short chain fatty acid

## Abstract

Pancreatic adenocarcinoma is one of the most lethal cancers in both men and women, with a median five-year survival of around 5%. Therefore, pancreatic adenocarcinoma represents an unmet medical need. Neoplastic diseases, such as pancreatic adenocarcinoma, often are associated with microbiome dysbiosis, termed oncobiosis. In pancreatic adenocarcinoma, the oral, duodenal, ductal, and fecal microbiome become dysbiotic. Furthermore, the pancreas frequently becomes colonized (by *Helicobacter pylori* and *Malassezia,* among others). The oncobiomes from long- and short-term survivors of pancreatic adenocarcinoma are different and transplantation of the microbiome from long-term survivors into animal models of pancreatic adenocarcinoma prolongs survival. The oncobiome in pancreatic adenocarcinoma modulates the inflammatory processes that drive carcinogenesis. In this review, we point out that bacterial metabolites (short chain fatty acids, secondary bile acids, polyamines, indole-derivatives, etc.) also have a role in the microbiome-driven pathogenesis of pancreatic adenocarcinoma. Finally, we show that bacterial metabolism and the bacterial metabolome is largely dysregulated in pancreatic adenocarcinoma. The pathogenic role of additional metabolites and metabolic pathways will be identified in the near future, widening the scope of this therapeutically and diagnostically exploitable pathogenic pathway in pancreatic adenocarcinoma.

## 1. Pancreatic Adenocarcinoma, an Unmet Medical Need

Pancreatic adenocarcinoma stems from the exocrine glands and ducts of the pancreas and usually appears in the head of the pancreas (2/3 of cases). Pancreatic adenocarcinoma is the fourth most prevalent cancer with the highest mortality in both men and women [1]. Worldwide, in 2018, 458,918 cases were reported, and 432,242 deaths were estimated to be linked to pancreatic adenocarcinoma [2]. The number of pancreatic adenocarcinoma cases has continued to rise [3] and is predicted to rise even more sharply in the future [4]. The five-year survival for pancreatic adenocarcinoma is around 5%, as the disease progresses asymptomatically to the locally advanced or metastatic stages, reducing therapeutic effectiveness [1]. Thus, late diagnosis and a low five-year survival rate represent an unmet medical need in pancreatic adenocarcinoma.

Curative surgical treatment can only be achieved in 15–20% of patients with pancreatic adenocarcinoma, due to the spreading of the disease around blood vessels, rendering patients inoperable. Neoadjuvant therapy can reduce tumor size and enable surgical excision. The chemotherapy regimen to combat pancreatic adenocarcinoma includes nucleoside analogs (gemcitabine, capecitabine), antimetabolites (5-fluorouracil), topoisomerase inhibitors (irinotecan), taxanes (Nanoparticle albumin-bound (NAB)-paclitaxel), and platinum compounds (oxaliplatin) [5]. Combinatorial chemotherapies are organized into regimens known as FOLFIRINOX (Folinic Acid-Fluorouracil-Irinotecan-Oxaliplatin) and FOLFOX (Folinic acid-Fluorouracil–Oxaliplatin) [5]. There are also new chemotherapy modalities on the way (e.g., Poly(ADP-ribose) polymerase (PARP) inhibitors [6]). Chemotherapy can be complemented by radiotherapy; however, evidence supporting the use of radiotherapy is very scarce. The treatment of pancreatic adenocarcinoma calls for a multidisciplinary approach especially in patients undergoing neoadjuvant therapy [7].

Environmental risk factors for pancreatic adenocarcinoma include smoking, alcoholism, chronic or recurrent pancreatitis, obesity, and diabetes mellitus [8]. Genetic mutations are also associated with pancreatic adenocarcinoma [9]. Mutations in *KRAS* were identified in approximately 80% of pancreatic adenocarcinoma cases [10]. Recent studies associated other mutations with pancreatic adenocarcinoma, including *BRCA1*, *TP53,* and a set of other DNA repair factors [9].

## 2. The Oncobiotic Transformation of the Microbiome

The microbiome shows characteristic changes in neoplastic diseases; the transformed microbiome, a characteristic of neoplasia, is termed the oncobiome [11,12,13,14,15,16,17,18]. Recent advances demonstrate that the oncobiome has a pathogenic role in neoplasia. An intricate relationship develops between the microbiome and the host, where the host can influence the composition and biomass of the microbiome through its behavior, feeding, and immune system, while the microbiome impacts on the host through secreting microbial metabolites, as well as serving as bait for the immune system [19,20,21,22,23,24,25,26,27,28,29,30].

Hanahan and Weinberg [31,32] coined the term “cancer hallmark”, which refers to a collection of biological processes that drive oncogenesis and support the unlimited proliferation of cancer cells. The oncobiome plays either a direct or tangential role in regulating all cancer hallmarks. The oncobiome is definitively involved in avoiding immune destruction, enhancing tumor promoting inflammation, activating movement, invasion, and metastasis, inducing angiogenesis, inducing genome instability and mutations, and deregulating cellular energetics [21,22,23,24,26,27,28,29,30,33,34,35].

Sustained inflammation and the consequent oxidative stress can lead to DNA damage and genomic instability, which are risk factors for accumulating mutations and, subsequently, for carcinogenic transformation [36,37,38]. A dysbiotic microbiome can drive local inflammation and, therefore, can be a driver of carcinogenesis, including pancreatic adenocarcinoma [39,40,41,42,43]. In contrast, increased oxidative stress can be cytostatic in certain malignancies, such as breast cancer [34,38]. In other words, oxidative stress, induced by oncobiosis, can induce malignancies, but in later stages can have cytostatic properties.

The oncobiome usually has a different immunogenic character than the normal microbiome (eubiome), as oncobiosis alters the immune system [21]. The tolerogenic character of the immune system inhibits the early elimination of cancer cells [21]. A more immunogenic microbiome supports immunotherapy/targeted therapy [27,44], while sustained, high-level inflammation can promote carcinogenesis [24,39,40,41,42,43]. In this process, the actual physical presence of the bacteria seems to be a key factor, but immunomodulatory bacterial metabolites are also important [23].

Several studies have shown that oncobiotic transformation supports cellular proliferation, invasion, and metastasis [22,23,24,25]. In addition, oncobiosis changes the expression of vascular-endothelial growth factor (VEGF) [23], implying that oncobiosis is involved in the regulation of tumor vascularization. To date, published studies show that these processes are the main targets of oncobiosis and oncobiotic bacterial metabolites.

What are the elementary steps behind these processes? Bacterial metabolites modulate the redox balance of cancer cells [24,34], as well as cancer cell metabolism [22,23]. These processes culminate in cytostasis, a reprogramming of the epithelial-mesenchymal transition leading to decreased cancer stem cells [22,23,24,25,29,34,35,45]. These basic events are the pillars for the inhibition of cancer cell growth, movement, and metastasis formation [46,47,48].

What can cause oncobiotic transformation or, in general, changes to the microbiome? Among the factors inducing oncobiosis, lifestyle plays a key role, including activities such as smoking [49], diet, obesity [50], changes to the diurnal rhythm [51,52,53], aging [54,55,56], underlying diseases such as diabetes [57], and exercise [58]. In fact, these factors are all individual risk factors for pancreatic adenocarcinoma. In cancers, other than pancreatic adenocarcinoma, antibiotic [59] and probiotic use [60,61] are also associated with carcinogenesis.

## 3. The Oncobiome in Pancreatic Adenocarcinoma

The relationship between the microbiome and pancreatic adenocarcinoma was first suggested by the discovery that *Helicobacter pylori* colonization was associated with pancreatitis [62]. This was followed by the discovery of associations between the oral [63], gut [64], pancreas [41,65,66], and fecal [43,67] microbiomes, the mycobiome [68], and pancreatic cancer. Since then, there has been an immense expansion of oncobiome studies focused on pancreatic adenocarcinoma.

According to our current understanding, elements of the oral, gastric, and intestinal microbiome can drive inflammation, which is a risk factor for carcinogenesis in the pancreas. In brief, the oral, gastric, and duodenal flora can colonize the common duct, the bile duct, and the pancreatic duct and, finally, the pancreas itself, as shown in a series of animal and human studies [39,64,66,69,70,71,72,73,74,75,76,77,78,79,80]. Characteristic changes occur to the oral [63,81,82,83,84,85,86,87,88] and duodenal microbiome [64] in pancreatic adenocarcinoma. *Enterobacter, Enterococcus, and E. coli* bactibilia [78] or the colonization of the pancreas [41,65,68,89,90,91,92,93,94] are risk factors for pancreatic adenocarcinoma. Of note, an oncogenic role of hepatotropic viruses (Hepatitis B and C virus and Transfusion Transmitted/Torque Teno virus) in pancreatic adenocarcinoma has been observed in a clinical setting, although the exact molecular mechanisms are yet unknown [78]. Similarly, changes to the mycobiome were also reported in pancreatic adenocarcinoma [68]. In a murine genetic model (*Kras*^−/−^ *Tp53*^−/−^ model), the food microbiome can also invade the pancreas [41]. The pathogenic role of bacterial invasion in the pancreas was demonstrated by the decreased incidence of pancreatic adenocarcinoma in gnotobiotic or antibiotic-treated mice [65]. Similar issues were raised in conjunction with human premedication before surgery in pancreatic carcinoma patients [92]. There seems to be a specificity among the antibiotics. For instance, penicillin increased the risk for pancreatic adenocarcinoma [95], while broad range antibiotic cocktails (streptomycin, gentamicin, bacitracin, and ciprofloxacin [65] or ampicillin, vancomycin, neomycin, and metronidazole [96]) were protective in murine models [97]. Furthermore, fecal microbiome transplantation modulates susceptibility to the disease [41,91].

The main findings concerning the oncobiome in pancreatic adenocarcinoma are summarized in Table 1. There is no consensus on how the diversity of the microbiome changes in pancreatic adenocarcinoma. The alpha diversity (Shannon index) of the tongue microbiome increases [86]. In contrast, the saliva microbiome showed no change in alpha diversity, while beta diversity was different between cases and controls [88]. There was a tendency towards a lower alpha index (Operational taxonomic unit (OTU) diversity) in the duodenum of pancreatic adenocarcinoma patients [64]. The alpha diversity (Chao1, Shannon) of the pancreatic microbiome differed between cases and controls, but the change was not consequent in the study of Pushalkar et al. [41] (also similar to the findings of [87] and [65]). The pancreatic mycobiome alpha diversity (OTU, Shannon) decreased [68] in pancreatic adenocarcinoma patients. Nevertheless, alpha diversity indices in patients with long-term survival were higher than short-term survival [91]. The alpha diversity of the stool microbiome in patients with adenocarcinoma was lower in two studies [43,94]. (For the explanation of the diversity indices we refer the reader to the following references [98,99]).

Some bacterial species showed a strong association with pancreatic adenocarcinoma. In the oral microbiome, *Porphyromonas gingivalis* increased in pancreatic adenocarcinoma [64,82,83,85,101]. *Helicobacter pylori* [62,69,70], *Enterobacter*, *Enterococcus* [64,90,92,93], *Fusobacteria* [89,94,102], and *E. coli* [64,93] were also shown to increase in pancreatic adenocarcinoma patients in multiple studies. In a study assessing intratumor DNA and serum cell-free DNA (1000+ patients), *Fusobacteria* count in tumors was higher compared to the healthy, untransformed tissues [102]. Fungal species, like *Malassezia,* also increased in pancreatic adenocarcinoma patients [68]. The oral microbiome can be used for diagnosis [63]. In fact, different risk factors of pancreatic adenocarcinoma are associated with changes to the microbiome, including smoking [103], poor oral health or tooth loss [78], or recurrent pancreatitis. There is a lower bacterial load in pancreatitis than in pancreatic adenocarcinoma.

Pancreatic bacterial invasion predominantly induces persistent inflammation. Both the innate and adaptive immunity participate in recognizing pancreatic bacteria and orchestrating the subsequent inflammatory reaction [39,40]. The involvement of Th1, Th2, and Th17 responses have all been demonstrated [41,42]. In pancreatic adenocarcinoma, the proportions of LPS-producing bacteria (e.g., *Prevotella*, *Hallella*, and *Enterobacter* [43]) increase. Lipopolysaccharide (LPS) can bind to the Toll-like (TLR) receptors; TLR2, TLR4, and TLR9 are associated with pancreatic adenocarcinoma development [104]. TLR activation induces the STAT3 and NF-κB pathways, which act as tumorigenic factors increasing cellular proliferation and suppressing apoptosis [39].

Besides the direct immunogenicity of the microbiome, an endocrine-like function was also described in several cancers [30,105,106,107] including pancreatic adenocarcinoma. Bacteria can produce bacterial metabolites that enter the systemic circulation and act on distant cancer cells. This process possesses features of endocrine signaling: a chemical entity is synthesized at one location, then transferred to another anatomical site where it binds to receptors and exerts biological responses there. Hereby, we will review the bacterial metabolites with possible pro- or anti-neoplastic features in pancreatic adenocarcinoma.

## 4. Bacterial Metabolites Playing Role in Pancreatic Adenocarcinoma

**Figure 1:**
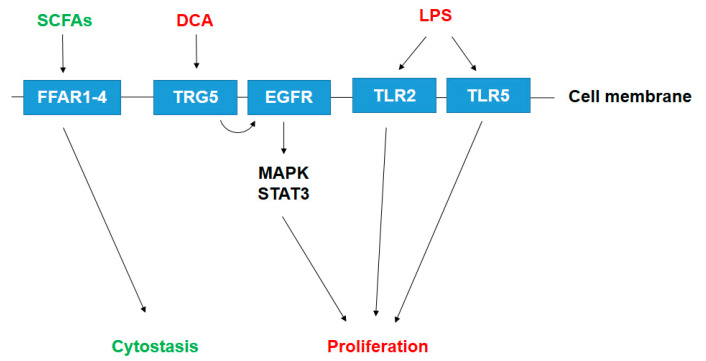
Known bacterial metabolite-elicited signaling pathways in pancreatic adenocarcinoma. Pro-proliferative metabolites are shown in red; antiproliferative metabolites are shown in green. Abbreviations: SCFA—short chain fatty acid, DCA—deoxycholic acid, LPS—lipopolysaccharide, FFAR—free fatty acid receptor, TGR5—Takeda G Protein-Coupled Receptor 5/ G-protein-coupled bile acid receptor, EGFR—Epidermal growth factor receptor, TLR—Toll-like receptor, MAPK—mitogen activated protein kinase, STAT—Signal transducer and activator of transcription.

The gut microbiome harbors a large number of species with an immense and diverse metabolism. Bacterial metabolites or components of bacteria can enter the systemic circulation of the host and be transferred to distant sites where the metabolites can exert hormone-like effects [19,30,108]. Bacterial metabolism is largely dysregulated in pancreatic adenocarcinoma [43]. Below, we will review the source and (possible) roles of pro- or anti-carcinogenic bacterial metabolites (Figure 1).

### 4.1. Short Chain Fatty Acids (SFCA)

Short chain fatty acids (SCFAs), namely acetate, propionate, butyrate, and lactate, are derived from non-digestible carbohydrates by bacterial saccharolytic fermentation [109,110]. The major SCFAs are acetate, propionate, and butyrate [111]. A smaller quantity of SCFA can be formed by amino acid deamination; this is the only source of branched-chain short chain fatty acids [110]. Hydrolysis, glycolysis, and the pentose-phosphate pathways are the key pathways for SCFA production [111], nevertheless, other pathways are also active. SCFAs are produced in the colon. SCFA production affects the pH of the colon and, hence, modulates the composition of the microbiome in the colon. SCFAs can reduce the proliferation of *Enterobacteriaceae* (e.g., *E. coli*, *Salmonella* ssp., or *Clostridia* ssp.) and *Borrelia burgdorferi* [112,113,114,115]. Furthermore, SCFAs can modulate the composition of the gut microbiome through the direct modulation of the immune system [116].

SCFA production is common among bacteria. *Bacteroidetes* primarily produce acetate and propionate, while *Firmicutes* chiefly produce butyrate [117]. *Akkermansia muciniphila* has a pivotal role in propionate synthesis through the degradation of mucin [118]. *Lachnospiraceae*, *Ruminococcus obeum,* and *Roseburia inulinivorans* produce propionate through the degradation of deoxy sugars (e.g., fucose, rhamnose), while *Bacteroidetes* and *Negativicutes* use hexoses to produce propionate [119]. Other propionate producers are *Phascolarctobacterium* spp., *Dialister* spp., *Veillonella* spp., *Salmonella* spp., *Megasphaera elsdenii*, and *Coprococcus catus* [120,121]. Acetate is predominantly produced by *Lactobacillus* spp., *Bifidobacterium* spp., *Akkermansia muciniphila*, *Bacteroides* spp., *Prevotella* spp., *Ruminococcus* spp., and *Streptococcus* spp [108]. The bulk of butyrate production can be linked to *Odoribacter, Anaeotruncus*, *Faecalibacterium prausnitzii,* *Eubacterium rectale*, *Roseburia faecis*, *Clostridium leptum*, *Coprococcus eutactus*, *Faecalibacterium prausnitzii*, *Eubacterium rectale, Anaerostipes caccae*, *Eubacterium hallii,* and an unnamed cultured species SS2/1 [120,121,122,123]. The bacterial species that produce bacterial metabolites are summarized in Table 2.

The human serum reference concentrations of SCFAs fall into the range of 10–100 µM [134,135,136]. However, local concentrations can be as high as 1 mM [137]. SCFAs primarily bind to the free fatty acid receptors (FFARs) found on both cancer cells and stromal cells [120,121,138,139]. SCFAs can be utilized as an energy source by cells [108] and SCFAs can modulate epigenetics through inhibiting histone deacetylases [140,141,142]. The activation of SCFA receptors controls numerous cancer hallmarks, including cell proliferation, apoptosis, cell invasion, gene expression, metabolism, and immune processes [140,141,142,143,144].

Acetate can ameliorate pancreatitis and its sequels, and, hence, protect against a risk factor of pancreatic adenocarcinoma [145]. Acetate drives the epigenetic reprogramming of mesenchymal stem cells towards cancer-associated fibroblasts that enhance the invasiveness of pancreatic adenocarcinoma cells [146]. Butyrate, at a 2 mM concentration, can reduce the proliferation of cultured pancreatic adenocarcinoma cells (Panc-1 and HPAF cells) and induce differentiation towards a secretory phenotype marked by ultrastructural changes [147]. Furthermore, a hyaluronic acid conjugate of butyrate proved to be cytostatic in a cultured pancreatic adenocarcinoma cell line [148]. Valproic acid, a branched chain synthetic SCFA, was also cytostatic in pancreatic adenocarcinoma cells when given in combination with 5-fluorouracil, suggesting similar properties for bacterial SCFAs [149]. In the pancreatic adenocarcinoma-associated oncobiome, probiotics and butyrate-producing bacteria decreased [43], suggesting that the above-detailed beneficial effects of SCFAs are largely suppressed in the disease.

### 4.2. Secondary Bile Acids

Chenodeoxycholic acid (CDCA) and cholic acid (CA) are primary bile acids, which are mainly synthesized in the liver; however, extrahepatic tissues (e.g., ovaries, macrophages, vascular endothelium, and brain) can contribute to this synthesis [150]. Primary bile acids are conjugated to glycine or taurine and are secreted into the bile, then, via bile, into the duodenum. Hepatic primary bile acids emulsify fats and activate lipases. The microbiome of the gut (mostly in the large bowels) dehydroxylate and deconjugate bile acids. Thus, primary bile acids are modified to produce secondary bile acids, including lithocholic acid (LCA), deoxycholic acid (DCA), and ursodeoxycholic acid (UDCA) [151]. There are 16 bile acids in early life, while in adulthood there are 20 different bile acids in humans [124,152,153]. The majority of bile acids undergo reuptake via the portal circulation and are then transported to the liver, where secondary bile acids are re-hydroxylated and re-conjugated for reuse. This cycle is called the enterohepatic circulation of bile acids. A small fraction of the reabsorbed bile acids can enter the systemic circulation [154] and systemic bile acids exert hormone-like, systemic effects [23,30,155,156,157].

The primary-to-secondary bile acid conversion is linked to the gut microbiome. Primarily, colonic bacteria are responsible for bile acid conversion; nevertheless, upper segments of the gastrointestinal tract may also play a role in bile acid transformation. Deconjugation takes place first, followed by oxidation, dehydroxylation, and epimerization. Deconjugation is catalyzed by bile salt hydrolases. *Bacteroides fragilis*, *Bacteroides vulgatus*, *Listeria monocytogenes*, *Clostridium*, *Lactobacillus*, and *Bifidobacterium* possess bile salt hydrolases [124,125,126,127,128,129]. Oxidation and epimerization activities are linked to intestinal *Firmicutes* (*Clostridium*, *Eubacterium*, and *Ruminococcus*), *Bacteroides*, and *Escherichia*, while dehydroxylation is linked to *Clostridia* and *Eubacteria* [124,125,126,128]. The enzymes involved in secondary bile acid production are assembled in the bile acid inducible (bai) operon in bacteria [151].

Bile acids have multiple receptors, including farnesoid-X-receptor (FXR), liver-X receptor (LXR), *Takeda* G Protein-Coupled Receptor 5/G-protein-coupled bile acid receptor (TGR5), constitutive androstane receptor (CAR), vitamin D receptor (VDR), and pregnane X receptor (PXR). These receptors are nuclear receptors, except for TGR5. Through these receptors, bile acids impact on immune responses, gastrointestinal mucosal barrier function, gestation [158], carcinogenesis [23,34], and metabolic diseases [155,159].

Bile acid homeostasis is largely hampered in cancer and metabolic diseases [160]. Originally, bile acids were regarded as procarcinogens [161]. However, recent advances suggest that some secondary bile acids can behave as both pro- and anti-carcinogens, depending on the cancer in question and the concentration of the bile acid present [23,34,105,107,162,163,164,165,166,167,168,169]. Bile acids also modulate the composition of the microbiome [160,170,171,172,173,174,175,176] and facilitate bacterial translocation into tissues [177], a key step in the carcinogenesis of pancreatic adenocarcinoma. Bacteria have different sensitivity for bile acids. *Enterococci* are considered bile acid resistant bacteria, which may explain the reports showing increased abundance of this bacteria in pancreatic adenocarcinoma [65,90,93].

Bile acid levels were reported to increase in pancreatic adenocarcinoma. A study comprised of 15 patients with pancreatic cancer and 15 patients with benign disease showed increasing trends in all bile acid species detected in pancreatic cancer patients. Increases in unconjugated bile acid levels in pancreatic adenocarcinoma patients were significant and surprisingly large (26 fold) [178].

Most bile acids have a carcinogenic role in pancreatic adenocarcinoma. Bile acids modulate risk factors for pancreatic adenocarcinoma; bile acids impact pancreatitis and bile acid efflux disorders, type II diabetes, obesity, and hyperlipidemia. Furthermore, bile acids reduce susceptibility to apoptosis, induce inflammatory mediators, and may perturb membranes and cellular movement (reviewed in [179]). Gallstones can obstruct the outflow of bile and, hence, can induce and sustain pancreatitis [180], a risk factor for pancreatic adenocarcinoma [179,181,182]. Exposure of pre-malignant pancreas ductal cells to bile may lead to carcinogenic transformation through inflammatory signaling, as demonstrated in rodent and human data [183,184,185,186]. DCA, through binding to TGR5, can activate EGFR, mitogen-activated protein kinase, and STAT3 signaling in pancreatic adenocarcinoma cells, inducing cell cycle progression [187]. Interestingly, there seems to be a selectivity among bile acids, as UDCA inhibits the epithelial-to-mesenchymal transition in pancreatic adenocarcinoma cell lines, and in that regard, acts as an anti-carcinogenic factor [188].

Expression levels for VDR [189], FXR [190], and PXR [191] are higher in tumor tissue than in the normal tissue of the pancreas. LXRβ, but not LXRα, is abundantly expressed in human pancreatic adenocarcinoma cases [192]. In the serum of PDAC patients, components of the LXR/RXR system are enriched [193]. Furthermore, higher FXR expression correlates with higher TNM stage, shorter survival, and poorer prognosis [190]. Higher PXR expression correlated with higher histological grade of pancreatic adenocarcinoma [191]. Nevertheless, unexpectedly, enhanced PXR/RXRβ expression correlated with smaller tumor size and the absence of lymph node metastases and longer survival [191]. Additionally, LXR agonist treatments disrupted proliferation, cell-cycle progression, and colony-formation in PDAC cells [194].

### 4.3. Polyamines

Polyamine metabolism is dysregulated in pancreatic adenocarcinoma [195]. The functional role of polyamine biosynthesis in (human) pancreatic adenocarcinoma is highlighted by the fact that the effects of the standard cytostatic therapies can be accentuated or ameliorated by modulation of the polyamine cycle [196,197,198,199,200]. Cadaverine, putrescine, spermine, and spermidine are classified as polyamines, but bacteria can produce other polyamines also [131,201].

Enzymes of the polyamine pathway had been identified in numerous species. However, the functional characterization of polyamine biosynthesis is limited to a few species [131]. *E. coli*, *Enterococcus faecalis*, *Staphylococcus aureus*, *Haemophilus influenzae*, *Neisseria flava*, *Pseudomonas aeruginosa*, *Campylobacter jejuni*, *Yersinia pestis*, *Vibrio cholerae*, *Bacteroides dorei*, *Bacteroides thetaiotaomicron*, *Bacteroides fragilis*, *Bacillus subtilis*, and *Proteus mirabilis* were shown to produce, accumulate, or need/use polyamines [108,130,131].

Cadaverine is a decarboxylation product of lysine and the bacterial enzymes LdcC and CadA are responsible for cadaverine biosynthesis [202,203]. Both the human body and bacteria can produce cadaverine. *Shigella flexneri*, *Shigella sonnei*, *Escherichia coli*, and *Streptococcus* possess enzymes for cadaverine biosynthesis [132]. Putrescine can be derived from arginine through decarboxylation, as is the case in *E. coli* [108]. Polyamines support bacterial growth and biofilm formation and in many pathogenic species are considered virulence factors [131].

A metabolomic and metatranscriptomic study of the fecal microbiome from a murine pancreatic adenocarcinoma model [67] showed that bacterial polyamine biosynthetic capacity was upregulated and aggravated by tumor progression. The main polyamines synthesized were putrescine, spermine, and spermidine. In accordance with these results, serum polyamine levels were also higher in pancreatic adenocarcinoma-bearing mice and patients. In contrast, Ren and co-workers [43] found that polyamine biosynthesis and transport pathways were downregulated in samples from pancreatic adenocarcinoma patients. Nevertheless, the lysine and putrescine transport systems were upregulated.

### 4.4. Bacterial Lipopolysaccharide (LPS)

Lipopolysaccharides, lypoglycans, or endotoxins are components of the bacterial outer membrane in Gram-negative bacteria [204,205]. Although LPS is not a classical bacterial metabolite in the strict sense, LPS seems to play a crucial role in the pathogenesis of pancreatic adenocarcinoma. Lipopolysaccharides are built upon a lipid anchor to which a polysaccharide chain is attached. The inherent role of LPS is to protect bacteria against toxins, antibiotics, or bile acids. However, LPS has high immunogenic potential and is considered a member of the pathogen associated molecular patterns (PAMPs). LPS elicits its effects through TLR4 and TLR2 receptors to induce innate immunity [204,205].

In pancreatic adenocarcinoma-associated oncobiosis, the proportion of LPS producing bacteria (*Prevotella*, *Hallella*, and *Enterobacter*) increases [43]. In addition, TLR2, TLR4, and TLR9 [104] and the downstream target of TLR4, MyD88 [40], are associated with pancreatic adenocarcinoma development. Taken together, LPS-induced TLR signaling likely plays a key role in maintaining inflammation in pancreatic adenocarcinoma.

### 4.5. Tryptophan Metabolites

Tryptophan is an amino acid with a very complex and intricate metabolism, in which bacterial metabolism plays a major role. A considerable portion of tryptophan, 4–6%, is metabolized by bacteria to yield indol derivatives [206]. In germ-free mice, serum tryptophan levels increase, emphasizing the volume of bacterial tryptophan degradation [133,207,208,209,210]. The bacterial metabolism of tryptophan has multiple branches [133,206], described as follows:

(1) The decarboxylation of tryptophan yields tryptamine. *Clostridium sporogenes* and *Ruminococcus gnavus* possess enzymes for tryptophan decarboxylation [133].
(2) Tryptophanase deaminates tryptophan to indole pyruvic acid, which is then metabolized to indole. Indole can be further oxidized and the subsequent conjugation of sulphate yields indican. Tryptophanase, denoted as TnaA, can be found in the tryptophanase operon [211]. Tryptophanase expression is widespread among bacteria [212,213].
(3) Indole pyruvic acid can be decarboxylated to indole acetaldehyde. Indole acetaldehyde can be converted to tryptophol or indole acetic acid. Indole acetic acid can be decarboxylated to yield skatole or conjugated with glutamine to yield indole acetic acid-glutamine. The main genera for this pathway are *Lactobacillus*, *Clostridium,* and *Bacteroides* [133].
(4) The reduction of indole pyruvic acid yields indole lactate, the dehydration of which yields indole acrylic acid. This compound can be reduced to indole propionic acid. Indole propionic acid can be further converted by human enzymes.

Tryptophan-derivatives (indoles) are ligands for the aryl hydrocarbon receptor (AHR) and can also bind to the PXR receptor [214,215,216]. AHR activation is a key element in the regulation of the immune system [133,217]. A tryptophan-poor diet has immunosuppressive effects in an AHR-dependent fashion [218]. By modulating mucosal immunity through AHR, indole derivatives influence the composition of the gut microbiome. For example, indole-derivatives can facilitate the expansion of *Lactobacillus reuteri* and inhibit the growth of pathogenic bacteria [214,219,220,221]. Furthermore, *Lactobacillus* utilizes tryptophan as an energy source [214].

Although direct data are missing for the effects of indole-derivatives in pancreatic adenocarcinoma, the invasive behavior of pancreatic adenocarcinoma cells can be modulated through the selective AHR modulators, Omeprazole and Tranilast [222]. Furthermore, as we noted above, a higher PXR expression correlates with a higher histological grade of pancreatic adenocarcinoma, while enhanced PXR/RXRβ expression correlates with a smaller tumor size, the absence of lymph node metastases, and longer survival [191].

### 4.6. Other Metabolites

To date, two studies reported in silico reconstruction of metabolic pathways of the microbiome in pancreatic adenocarcinoma. These data identify bacterial metabolites that potentially influence pancreatic adenocarcinoma cells or carcinogenesis itself, thus, we review these data and provide a list in Table 3.

Mendez and colleagues [67] reported a time course experiment using a murine model of pancreatic adenocarcinoma. The results of this experiment revealed that the microbiome in pancreatic adenocarcinoma shifted towards nucleotide, lipid, and polyamine biosynthesis that was accentuated during the progression of the disease. Increased polyamine biosynthesis was confirmed by direct measurement of polyamines in the serum of tumor-bearing mice and healthy controls and pancreatic adenocarcinoma patients. In addition, hexitol fermentation, carbohydrate metabolism, and vitamin biosynthesis and metabolism were upregulated.

The second study was a prospective study of 85 pancreatic adenocarcinoma patients and 57 matched healthy controls in which the fecal microbiome was assessed [43]. A decrease was observed in various transport systems, amino acid metabolism, and core metabolic pathways in pancreatic adenocarcinoma patients. Among the upregulated pathways were genes for amino acid metabolism, carbohydrate metabolism, transport systems, and metabolic pathways.

## 5. Supporting Clinical Decision Making, Diagnostic Applications

As we noted earlier, certain studies assessing the oncobiome in pancreatic adenocarcinoma came to the conclusion that the composition of the oral [63,82,86,88,223], gut [43], pancreatic [102], and fecal [100] microbiomes differ from the corresponding healthy microbiomes. Thus, the microbiomes can be used for diagnostic applications. Diagnostics can be useful for the assessment of the risk for tumor development (i.e., tumor detection) [82], survival prediction [41,91], deducting etiology [100], predicting mortality [89], and selecting between the forms of the disease (e.g., obstructive versus non-obstructive forms [43]).

Most studies mentioned above had low patient numbers (tens of patients to a few hundred) and, therefore, did not reach the level of statistical significance. When already existing shotgun sequencing data of tumor DNA was assessed for the presence of bacterial DNA detected statistically [102], counts of bacteria in tumors differed from the corresponding non-transformed tissues. Importantly, patterns were identified that had suitable specificity and selectivity values for subsequent diagnostic applications. Furthermore, these patterns were detectable in serum in the form of cell-free DNA, suggesting that serum could be used for diagnostic applications [102]. In addition, oral swab or feces can be used as easily accessible biomaterials for the detection of pancreatic adenocarcinoma [82,88,100].

As the detection of pancreatic adenocarcinoma is difficult at early (curable) stages, early detection through detecting oncobiotic transformation has clear advantages for patients. The choice of antibiotics used for premedication before surgical excision of pancreatic adenocarcinoma can also be based on the composition of the pancreatic microbiome [92].

## 6. Future Directions

**Figure 2:**
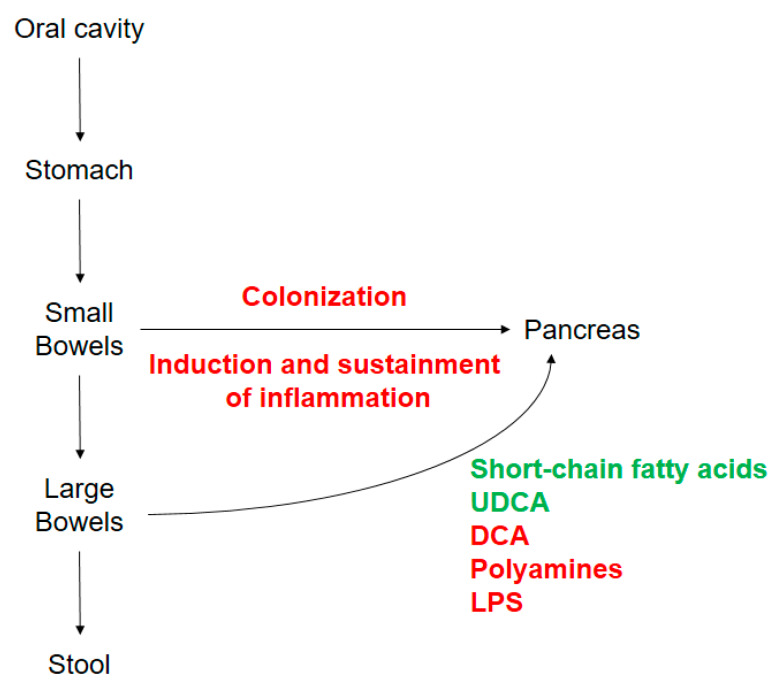
Schematic representation of the role of oncobiosis in pancreatic adenocarcinoma. Rows represent the spillover of the dysbiotic microbiome of the oral cavity, stomach, and bowels to the pancreas and feces. Antineoplastic processes are shown in green and neoplastic processes are shown in red. Abbreviations: UDCA—ursodeoxycholic acid, DCA—deoxycholic acid, LPS—lipopolysaccharide.

Oncobiosis in pancreatic adenocarcinoma is a complex process, involving multiple microbiome compartments, including the oral, gastric, duodenal, ductal, pancreatic, and fecal compartments. The bacterial colonization of the pancreas drives inflammation and probably facilitates the initiation and progression of the disease to determine the aggressiveness of the disease. Furthermore, there seems to be a large set of bacterial metabolites released into the circulation or tumor microenvironment that has direct effects on the behavior of pancreatic adenocarcinoma cells (Figure 2).

The involvement of bacterial metabolites is just coming of age in the microbiome field. In other cancers, the involvement of the microbiome has gained ground quickly and holds promise for new treatment modalities [224,225]. Studies on the in silico reconstruction of microbiome metabolism and other circumstantial data suggest large changes to the bacterial metabolome, making it likely that such metabolites will be identified and characterized in the future. It is important to note that in studies with bacterial metabolites, metabolites must be used in concentrations corresponding to serum or tissue reference concentrations to avoid the non-physiological effects of supraphysiological concentrations.

Establishing the causative role of oncobiosis in pancreatic adenocarcinoma will facilitate the assessment of how antibiotics, probiotics, or prebiotics may modulate the behavior of the disease in analogy to other diseases and studies [59,60,226,227,228]. Dietary intervention or special diets can be proposed to patients [229]. Thus, the study of the microbiome may change personalized medicine [100]. A better understanding of the oncobiome in pancreatic adenocarcinoma holds the promise of prolonging survival in pancreatic adenocarcinoma.

## Figures and Tables

**Table 1:** The main findings of the human oncobiome studies in pancreatic adenocarcinoma.

Sample Type and Sample Size	Method	Changes to Microbiome	Other Observations	Ref.
**Changes to the oral microbiome**				
11,328 individuals in a prospective study. Dental health was monitored between 1971–1992			Periodontitis increases the risk for pancreatic adenocarcinoma.	[81]
10 resectable patients with pancreatic cancer and 10 matched healthy controls for oral microbiome assay, 28 resectable pancreatic cancer, 28 matched healthy controls, and 27 chronic pancreatitis samples for validation	HOMIM hybridization array	*Streptococci, Veilonella, Actinobacteria, Campylobacter,* and *Prevotella* increased in pancreatic adenocarcinoma patients.	*Neisseria elongata* and *Streptococcus mitis* were validated as biomarkers for pancreatic adenocarcinoma.	[63]
Pre-diagnosis blood samples from 405 pancreatic cancer cases and 416 matched controls, collected as part of the European Prospective Investigation into Cancer and Nutrition study			High serum antibodies against *Porphyromonas gingivalis* ATTC 53978 showed a two fold increase in risk for pancreatic adenocarcinoma. Those individuals who had high antibody titer against the commensal flora had a lower risk for pancreatic adenocarcinoma as compared to those with low titer.	[82]
8 pancreatic adenocarcinoma patients and 22 healthy controls	16S rDNA was amplified and sequenced	The pancreatic cancer group had higher levels of *Leptotrichia*, and lower levels of *Porphyromonas*, and *Neisseria.* No difference in diversity. *Leptotrichia* to *Porphyromonas* ratio was significantly higher in pancreatic adenocarcinoma patients.		[83]
Among 149 orodigestive cancers 6 pancreatic adenocarcinoma cases			*Treponema denticola* chymotrypsin-like proteinase that can induce matrix metalloproteinases, was found in pancreatic adenocarcinoma using immunohistochemistry.	[84]
361 incident pancreatic adenocarcinoma patients and 371 matched controls from two prospective cohort studies, the American Cancer Society Cancer Prevention Study II and the National Cancer Institute Prostate, Lung, Colorectal and Ovarian Cancer Screening Trial.	DNA was isolated from oral wash samples; 16S rRNA gene V3-V4 was amplified and sequenced using Roche 454 FLX Titanium Pyrosequencing system	Carriage of *Porphyromonas gingivalis* and *Aggregatibacter actinomycetemcomitans* were associated with a higher risk for pancreatic adenocarcinoma. *Fusobacteria* and its genus, *Leptotrichia* decreased the risk of pancreatic adenocarcinoma. The genus *Alloprevotella* increased the risk for pancreatic adenocarcinoma.		[85]
30 stage I pancreas head adenocarcinoma patients and 25 healthy controls	Tongue scrapes were collected and the V3-V4 16S rDNA was amplified and sequenced	*Leptotrichia*, *Fusobacterium*, *Rothia*, *Actinomyces*, *Corynebacterium*, *Atopobium*, *Peptostreptococcus*, *Catonella*, *Oribacterium*, *Filifactor*, *Campylobacter*, *Moraxella,* and *Tannerella* were overrepresented, while *Haemophilus*, *Porphyromonas,* and *Paraprevotella* were underrepresented in pancreatic adenocarcinoma patients.	*Haemophilus*, *Porphyromonas*, *Leptotrichia,* and *Fusobacterium* distinguished pancreatic adenocarcinoma patients from healthy subjects.	[86]
Saliva samples from 280 pancreatic adenocarcinoma cases (29 stage I, 160 stage II, 37 stage III, and 54 stage IV pancreatic tumors) of which 273 was used in the study and 285 controls	V4 region of the 16S rRNA gene was PCR amplified and sequencing was performed on the Illumina MiSeq.	*Haemophilus* genus showed a marginal association with pancreatic cancer risk. *Enterobacteriaceae*, *Lachnospiraceae*, *Bacteroidaceae*, and *Staphylococcaceae* showed a positive correlation with pancreatic cancer risk.		[88]
***Helicobacter pylori* colonization (seropositivity)**				
Cases with pancreatic cancer (*n* = 87) were matched to controls (*n* = 263) using age, sex and time for baseline investigation as matching variables	*H.pylori* serology was analyzed in stored serum samples using an enzyme-linked immunosorbent assay		*H.pylori* seropositivity was not associated with pancreatic cancer in the total cohort (adjusted OR 1.25 (0.75–2.09)). However, a statistically significant association was found in never smokers (OR 3.81 (1.06–13.63) adjusted for alcohol consumption) and a borderline statistically significant association was found in subjects with low alcohol consumption (OR 2.13 (0.97–4.69) adjusted for smoking).	[62]
110 patients with pancreatic cancer	A polypeptide antibody against the plasminogen-binding protein (PBP) of *Helicobacter pylori* and with the ubiquitin-protein ligase E3 component, n-recognin 2 (UBR2), an enzyme highly expressed in acinar cells of the pancreas		The antibody was positive in 5 of 110 patients with pancreatic cancer (5%).	[69]
Venipuncture specimens were obtained from a representative sample of 761 case patients and 794 randomly selected control subjects matched by category of age and gender	Antibody seropositivity for *H.* *pylori* and its virulence protein CagA were determined by commercial enzyme-linked immunosorbent IgG assays		Compared with individuals seronegative for both *H.* *pylori* and CagA, decreased pancreas-cancer risk was seen for CagA seropositivity [adjusted OR, 0.68; 95% confidence interval (CI), 0.54–0.84], whereas some increased risk was suggested for CagA-negative *H.* *pylori* seropositivity (OR, 1.28; 95% CI, 0.76–2.13).	[70]
**Changes to the duodenal microbiome**				
14 patients with pancreatic head cancer and 14 healthy controls. Endoscopic duodenal mucosal biopsies	16S rRNA gene pyrosequencing after the PCR amplification of the V3-V4 region. The rarefaction curves did not approach a plateau.	*Acinetobacter*, *Aquabacterium*, *Oceanobacillus*, *Rahnella*, *Massilia*, *Delftia*, *Deinococcus*, and *Sphingobium* were more abundant in the duodenal mucosa of pancreatic cancer patients, whereas the duodenal microbiomes of healthy controls were enriched with *Porphyromonas*, *Paenibacillus*, *Enhydrobacter*, *Escherichia*, *Shigella*, and *Pseudomonas*. Alpha and beta diversity were not different between the two groups.	Pancreatic adenocarcinoma patients have a higher incidence of *H. pylori* colonization.	[64]
**Changes to the pancreatic microbiome**				
283 patients with pancreatic ductal adenocarcinoma (PDAC)	Genomic DNA extracted from FFPE tissue specimens assessed using TaqMan primer/probe sets to detect *Fusobacterium* species		8.8% detection rate of *Fusobacterium* species in pancreatic cancers; however, tumor *Fusobacterium* status was not associated with any clinical and molecular features. In multivariate Cox regression analysis, compared with the *Fusobacterium* species-negative group, higher cancer-specific mortality rates were observed in the *Fusobacterium* positive group.	[89]
Human FFPE pancreatic adenocarcinoma samples (*n* = 27)	Illumina sequencing of V1-V3 hypervariable regions of 16S RNA gene.	Differential presence of *Acinetobacter, Afipia, Corynebacterium, Deltia, Enterobacter, Enterococcus, Escherichia, Klebsiella, Propionibacterium, Pseudomonas, Rastoria, Sphingomonas, Staphylococcus,* and *Streptococcus* between healthy, pancreatitis, and pancreatic adenocarcinoma tissues.	In the pancreas, the microbiome could not discriminate between healthy, pancreatitis, and pancreatic adenocarcinoma states. Culturable bacteria are present in the human pancreas with a mean of ~1 × 10^5^ (aerobic) and ~1 × 10^5^ (anaerobic) cfu/g of tissue after 48 h of culture.	[65]
Pancreatic juice from pancreatic cancer (*n* = 20) and duodenal cancer/bile duct cancer (*n* = 16) patients	PCR identification of bacterial species by 16S ribosomal RNA gene.	*Enterococcus faecalis* may be involved in pancreas carcinogenesis.	*E. faecalis* is present in pancreas tissue in cancer patients. Antibodies against *E. faecalis* capsular polysaccharide is elevated in chronic pancreatitis patients.	[90]
Patients with pancreatic adenocarcinoma (*n* = 32), matched healthy individuals (*n* = 31). stool and pancreas tissue were assessed	Sequencing of the V3-V4 hypervariable region of the 16S RNA gene after PCR amplification	*Proteobacteria* are more abundant in patients with pancreatic adenocarcinoma as compared to gut cancer patients.		[41]
105 subjects were enrolled of which 27 had pancreatic adenocarcinoma, 57 had intraductal papillary mucinous neoplasms and 21 had benign lesions	Pancreas cyst fluid was collected, total bacterial 16S copy number was assessed, and 16S DNA was sequenced	*Fusobacterium nucleatum* and *Granulicatella adiacens* were associated with high-grade dysplasia. In network analysis, the network’s nodes were *Actinobacteria* (*Cutibacterium acnes*), *Bacteroidetes*, *Firmicutes* (*Streptococcus anginous*, *Granulicatella adiacens*), and *Proteobacteria* (*Klebsiella aerogenes*).	The number of 16S reads increases in precancerous and cancer cases.	[87]
Human fecal samples and specimens of pancreatic tissue were collected under sterile conditions from healthy volunteers and patients undergoing surgery for PDA or pancreatic endocrine tumors (benign disease)	PCR amplification and sequencing of the ITS1 region of the 18S rRNA gene using Illumina sequencing	Pancreatic adenocarcinoma tumors with fungal infiltration were enriched for *Malassezia* spp.	Ligation of mannose-binding lectin (MBL), which binds to glycans of the fungal wall to activate the complement cascade, was required for oncogenic progression.	[68]
Long-term surviving (*n* = 22) and short-term surviving (*n* = 21) pancreatic adenocarcinoma patients.	From the tumor and feces 16S rDNA V4 region was amplified by PCR and sequenced in the MiSeq platform (Illumina).	Intratumoral microbiome signature (*Pseudoxanthomonas*-*Streptomyces*-*Saccharopolyspora*-*Bacillus clausii*) is highly predictive of long-term survivorship.	The microbiome that provides long-term survival can be transplanted.	[91]
50 patients with pancreatic adenocarcinoma were enrolled. In cases where a biliary stent was inserted prior to surgery, the stent was removed and cultured. In other cases, swabs of bile or pancreatic fluid and tissue from the bile duct or pancreas were obtained and cultured.	Classical culture	96% of the specimens demonstrated the presence of microbes, 90% of all cases were polymicrobial. The most frequent species found were *Enterobacteriaceae*, *Enterococcus* species, *Candida* species, and *Streptococcus milleri*		[92]
152 Italian patients of which 72 had pancreas head adenocarcinoma patients were present	Classical culture	The most common bacteria among pancreas head adenocarcinoma patients were *E. coli*, *K. pneumoniae*, and *P. aeruginosa*, and less frequently, *Alcaligenes* spp., *Serratia* spp., and *Enterococcus* spp.	Although pancreas head carcinoma patients were not assessed separately, only such patients were present in the shortest survival cohort enabling the assessment of that patient population. *E. coli*, *K. pneumoniae*, and *P. aeruginosa* showed a high percentage of resistance to third-generation cephalosporins (3GCs), aminoglycosides class, and quinolone group, especially to levofloxacin, but the same bacteria were sensitive to carbapenems.	[93]
50 patients with pancreatic adenocarcinoma, 34 other organs (i.e., controls). In total, 189 tissue samples (pancreatic duct, duodenum, pancreas), 57 swabs (bile duct, jejunum, stomach), and 12 stool samples.	The 16S rRNA V3–V4 hypervariable regions were amplified using Illumina MiSeq	*Lactobacillus* ssp. was significantly higher in noncancer subjects compared with cancer subjects and the relative abundance of *Fusobacterium* spp was higher in cancer subjects compared with noncancer subjects.		[94]
**Changes to stool microbiome**				
Prospective study, 85 pancreatic cancer (PC) and 57 matched healthy controls (HC)	MiSeq sequencing	Phylum *Bacteroidetes* was significantly increased, while *Firmicutes* and *Proteobacteria* were decreased in PC patients versus healthy controls.	Gut microbial diversity decreased in pancreatic adenocarcinoma. Alpha diversity decreased. The abundance of certain pathogens and lipopolysaccharides-producing bacteria increased. Probiotics and butyrate-producing bacteria decreased. Changes to the microbiome can be used as markers to detect pancreatic adenocarcinoma and the obstructive and non-obstructive forms.	[43]
Patients with pancreatic adenocarcinoma (*n* = 32), and matched healthy individuals (*n* = 31). stool and pancreatic tissue were assessed	Sequencing of the V3-V4 hypervariable region of the 16S RNA gene after PCR amplification	*Proteobacteria* are more abundant in patients with pancreatic adenocarcinoma as compared to healthy controls.		[41]
Long-term surviving (*n* = 22) and short-term surviving (*n* = 21) patients. Sequencing of intratumor and stool microbiomes.	16S rDNA V4 region was amplified by PCR and sequenced in the MiSeq platform (Illumina).	Intra-tumoral microbiome signature occurs in pancreatic adenocarcinoma patients (*Pseudoxanthomonas*-*Streptomyces*-*Saccharopolyspora*-*Bacillus clausii*) that is highly predictive of long-term survivorship.	The microbiome that provides long-term survival can be transplanted.	[91]
30 patients with pancreatic adenocarcinoma, 6 patients with pre-cancerous lesions, 13 healthy subjects, and 16 with non-alcoholic fatty liver disease	16S RNA was PCR amplified and was sequenced using the Illumina MiSeq platform and LEfSe linear discriminant analysis (LDA) was performed		Patterns of the microbiome can separate pancreatic adenocarcinoma patients from healthy subjects and patients with comorbidities (NAFLD, etc.) and can discriminate between the etiology of pancreatic adenocarcinoma.	[100]

**Table 2:** The microbial source of the metabolites mentioned in the review.

Parent Metabolite	Bioactive Metabolite	Genus	Species	Reference
complex carbohydrates simple carbohydrates mucin	acetate	*Lactobacillus*		[108]
		*Bifidobacterium*		[108]
		*Bacteroides*		[108]
		*Prevotella*		[108]
		*Ruminococcus*		[108]
		*Streptococcus*		[108]
	propionate	*Lachnospiraceae*		[119]
		*Bacteroidetes*		[119]
		*Negativicutes*		[119]
		*Phascolarctobacterium*		[120,121]
		*Dialister*		[120,121]
		*Veillonella*		[120,121]
		*Salmonella*		[120,121]
			*Megasphaera elsdenii*	[120,121]
			*Coprococcus catus*	[120,121]
			*Akkermansia muciniphila*	[118]
			*Ruminococcus obeum*	[119]
			*Roseburia inulinivorans*	[119]
	butyrate	*Odoribacter*		[120,121,122,123]
		*Anaeotruncus*		[120,121,122,123]
			*Faecalibacterium prausnitzii*	[120,121,122,123]
			*Eubacterium rectale*	[120,121,122,123]
			*Roseburia faecis*	[120,121,122,123]
			*Clostridium leptum*	[120,121,122,123]
			*Coprococcus eutactus*	[120,121,122,123]
			*Faecalibacterium prausnitzii*	[120,121,122,123]
			*Eubacterium rectale*	[120,121,122,123]
			*Anaerostipes caccae*	[120,121,122,123]
			*Eubacterium hallii*	[120,121,122,123]
			unnamed cultured species SS2/1	[120,121,122,123]
primary bile acids	secondary bile acids	*Clostridium*		[124,125,126,127,128,129]
		*Lactobacillus*		[124,125,126,127,128,129]
		*Bifidobacterium*		[124,125,126,127,128,129]
		*Eubacterium*		[124,125,126,128]
		*Ruminococcus*		[124,125,126,128]
		*Escherichia*		[124,125,126,128]
			*Bacteroides fragilis*	[124,125,126,127,128,129]
			*Bacteroides vulgatus*	[124,125,126,127,128,129]
			*Listeria monocytogenes*	[124,125,126,127,128,129]
	polyamines		*E. coli*	[108,130,131]
			*Enterococcus faecalis*	[108,130,131]
			*Staphylococcus aureus*	[108,130,131]
			*Haemophilus influenzae*	[108,130,131]
			*Neisseria flava*	[108,130,131]
			*Pseudomonas aeruginosa*	[108,130,131]
			*Campylobacter jejuni*	[108,130,131]
			*Yersinia pestis*	[108,130,131]
			*Vibrio cholerae*	[108,130,131]
			*Bacteroides dorei*	[108,130,131]
			*Bacteroides thetaiotaomicron*	[108,130,131]
			*Bacteroides fragilis*	[108,130,131]
			*Bacillus subtilis*	[108,130,131]
			*Proteus mirabilis*	[108,130,131]
lysine	cadaverine	*Streptococcus*		[132]
			*Shigella flexneri*	[132]
			*Shigella sonnei*	[132]
			*Escherichia coli*	[132]
tryptophan	tryptamine		*Clostridium sporogenes*	[133]
			*Ruminococcus gnavus*	[133]
	indole acetic acid	*Lactobacillus*		[133]
		*Clostridium*		[133]
		*Bacteroides*		[133]

**Table 3:** Bacterial metabolic pathways dysregulated in pancreatic adenocarcinoma.

Study	Direction of Regulation in Patients	Biological Process	Biochemical Process
[67]	upregulated		Nucleotide biosynthesis
			Lipid biosynthesis
			Polyamine biosynthesis
			Hexitol fermentation
			Carbohydrate metabolism
			Vitamin biosynthesis and metabolism
[43]	downregulated	transport systems	Phosphate transport system (M00222)
			Cobalt transport system (M00245)
			Mannopine transport system (M00301)
			Glutamate transport system (M00233)
			Trehalose-maltose transport system (M00204)
			Spermidine-putrescine transport system (M00299)
		amino acid metabolism	Histidine biosynthesis (M00026)
			Glutamate transport system (M00233)
		metabolic pathways	Complex I/NADH dehydrogenase (M00144)
			Pentose phosphate pathway/non-oxidative phase (M00007)
			V type ATPase (M00159)
			Pyrimidine deoxyribonucleotide biosynthesis (M00053)
			Pyruvate ferredoxin oxidoreductase (M00310)
	upregulated	amino acid metabolism	Leucine biosynthesis (M00019)
			Twin arginine translocation/Tat system (M00336)
			Histidine degradation (M00045)
			Methionine salvage pathway (M00034)
			Lysine arginine ornithine transport system (M00225)
			Dipeptide transport system (M00324)
			Arginine transport system (M00229)
			Histidine transport system (M00226)
		carbohydrate metabolism	Oligogalacturonide transport system (M00202)
			Entner Doudoroff pathway (M00008)
		transport systems	Putative spermidine putrescine transport system (M00193)
			Microcin C transport system (M00349)
			Putrescine transport system (M00300)
			Sec secretion system (M00335)
			Histidine transport system (M00226)
		metabolic pathways	Pyridoxal biosynthesis (M00124), Citrate cycle (M00011)
			Complex II/succinate dehydrogenase (M00150)
			Glyoxylate cycle (M00012)
			C5 isoprenoid biosynthesis/non-mevalonate pathway (M00096)
			Ubiquinone biosynthesis (M00117)
			Prokaryotic GABA biosynthesis (M00136)
			Lipopolysaccharide biosynthesis (M00060)
			Bacterial DNA polymerase III complex (M00260)
		polyamine biosynthesis and transport	Polyamine biosynthesis (M00133)
			Spermidine putrescine transport system (M00299)
